# Volumetric efficiency in motor-driven two-dimensional piston pumps with leakage and reverse flow under high pressure

**DOI:** 10.1038/s41598-024-79700-9

**Published:** 2024-11-22

**Authors:** Yong Chen, Congcong Hua, Chengwei Tong, Yiren Zang, Jian Ruan

**Affiliations:** 1 School of Mechanical Engineering, Zhejiang University of Technology, Hangzhou, 310023 China; 2 Shenhao Technology Co., Ltd., Hangzhou, 311121 China

**Keywords:** Reverse flow, Hydraulic pump, Motor-driven pump, Two-dimensional piston pump, Volumetric efficiency, Engineering, Mechanical engineering

## Abstract

This paper presents a volumetric efficiency model for high-pressure motor-driven 2D piston pumps, incorporating key factors such as axial internal and external leakage, circumferential leakage, and reverse flow. The model integrates hydraulic fluid compressibility and variations in flow coefficients to improve simulation accuracy. Co-simulation using AMESim and Simulink, along with experimental validation, demonstrates the model’s reliability across various operating conditions. The results show that rotational speed enhances volumetric efficiency due to its minimal effect on leakage and reverse flow, while pressure significantly reduces efficiency, particularly through reverse flow and circumferential leakage. Reducing trapped volume proves to be an effective strategy for minimizing reverse flow and improving overall pump performance. Additionally, refining flow coefficients to improve the accuracy of the simulation model remains an important area for further development.

## Introduction

Since the adoption of the Paris Agreement in 2015^1^, there has been a global commitment to limit the increase in global temperatures to well below 2 °C above pre-industrial levels, with a targeted effort to restrict the rise to 1.5 °C. Achieving this ambitious target necessitates the widespread adoption of technologies and practices across industries that enhance energy efficiency and mitigate greenhouse gas emissions. Traditional hydraulic systems, which commonly utilize internal combustion engines, are less efficient and generate higher levels of pollutants compared to electric motor-driven systems. A transition towards motor-driven pumps (MDPs) within hydraulic systems presents a significant opportunity to reduce both energy consumption and emissions.

Currently, the electrification of axial piston pumps is a major focus in the high-power MDP sector, emerging as a hot topic within the industry^2^. However, this electrification effort faces numerous challenges. The design of lubrication pairs in these pumps typically requires balancing factors related to load and speed^3–5^. Under motor-driven applications, which often involve a wide range of variable speed and pressure conditions, along with loaded startups, these friction pairs are subjected to harsh operating environments, increasing the risk of stiction.

To overcome these challenges, various new pump designs have been proposed. INNAS^6^ has introduced a novel floating cup axial piston pump. It features a mirror-symmetrical design with 12 pistons on each side, totaling 24 pistons, which significantly reduces axial force and bearing load compared to the traditional odd-numbered piston design. This also effectively reduces pulsation and fluid noise^7^. The distribution plate has a small tilt angle, around 8–12°, and eliminates the need for a slipper structure, making it more conducive to loaded startup and resulting in a more compact structure. The pistons are fixed while the floating cup reciprocates relative to the pistons, causing volume changes. The spherical piston heads form a sealing line with the floating cup, ensuring good sealing performance under high pressure^8^.

Another important advancement is the Digital Displacement Pump (DDP) by Danfoss^9^. This pump achieves higher efficiency and better control performance by precisely controlling the opening and closing of each piston. Unlike traditional hydraulic pumps, the DDP uses an electronic control system to adjust displacement in real-time to optimize energy use, thus improving system efficiency. The pump’s design features a multi-piston structure, with each piston controlled by an independent solenoid valve, allowing dynamic participation based on demand. Experimental data shows that the DDP can achieve an overall efficiency of over 85%, with a maximum of 89% under various operating conditions. With rapid response characteristics, the pump quickly adapts to load changes, significantly enhancing productivity and operational precision, while providing excellent dynamic response and controllability^10^.

These two designs effectively reduce driving power and energy consumption in engineering machinery while achieving high startup efficiency, making significant contributions to the electrification of hydraulic systems. Another approach was introduced by Ruan et al.^11^, who developed the 2D piston pump. This pump uses two pairs of roller-cam mechanisms for the rotation-to-reciprocation conversion, eliminating the need for oil film lubrication and allowing it to adapt to transient operating conditions and loaded startups. The piston shaft performs both reciprocating motion and distribution functions, an integrated design that enhances the power-to-weight ratio. As a result, this structure offers space-saving and weight-reduction benefits, making it particularly suitable for aerospace^12^ and robotics applications.

Volumetric efficiency, defined as the ratio of actual to theoretical output flow, is a critical factor in hydraulic pump performance. Improving volumetric efficiency not only conserves energy but also extends the operational range. Given the similarities between the 2D piston pump and axial piston pumps, the following research offers valuable insights for the 2D pump design. Volumetric losses in axial piston pumps result from gap leakage and compression losses.

Leakage commonly occurs at interfaces like the valve plate/cylinder block, piston/cylinder and piston/slipper. Ivantysynova et al.^13,14^ developed non-isothermal gap flow simulations to predict leakage, while CASPAR software optimized valve plate designs, incorporating features such as precompression grooves and cross ports^15,16^. Wang^17^ refined valve plate geometry using pressure carryover concepts to reduce energy losses, and Manring^18^ further improved volumetric efficiency by optimizing sealing land geometry and reducing leakage using Poiseuille’s law, particularly at higher pressures.

Compression losses, influenced by pressure, oil viscosity, and aeration, significantly impact volumetric efficiency. Paszota^19^ introduced a model for evaluating these losses. Cavitation, in particular, exacerbates compression losses, as confirmed by Geng et al.^20^, who identified nonlinear compression losses due to air content. Qing et al.^21^ further demonstrated that cavitation plays a major role in these losses, especially at high rotational speeds, with compression losses potentially constituting over 50% of total volumetric losses under specific conditions. Park et al.^22^ found that increasing suction pressure helps mitigate cavitation and reverse flow, thereby improving volumetric efficiency.

Kauranne et al.^23^ also noted that efficiency decreases with rising pressure but improves with increased rotational speed and derived capacity. Frosina et al.^24^ found that incorporating pressure relief grooves and a pre-compression filter volume (PCFV) in axial piston pumps smooths pressure transitions, reducing reverse flow and enhancing efficiency. Zaluski^25^ demonstrated that shifting the swash plate’s axis reduces dead space, thereby minimizing compression losses. Additionally, Zaluski^26^ further demonstrated that shifting the swash plate’s axis of rotation improves volumetric efficiency, particularly at high pressures, leading to up to a 15% reduction in volumetric losses.

Huang et al.^27^ investigated 2D piston pumps operating at pressures up to 8 MPa, focusing on how gap flow changes with pressure in low-viscosity fluids, such as aviation fuel. Their study revealed that as pressure increases, volumetric losses rise almost linearly due to gap leakage, with minimal influence from rotational speed, which results in higher volumetric efficiency at greater speeds. Although they mentioned reverse flow, its impact was not fully examined or compared to leakage, particularly under higher pressure conditions.

Building upon previous research on piston pumps, this study focuses on developing a more precise model by accounting for key factors such as fluid compressibility and variations in flow coefficients at distribution windows, to better reflect the operational conditions of higher viscosity fluids and elevated load pressures in electric hydraulic pump systems. Based on this model, methods to reduce reverse flow are proposed to enhance volumetric efficiency.

## Structure and principle

### Structure

**Fig. 1:**
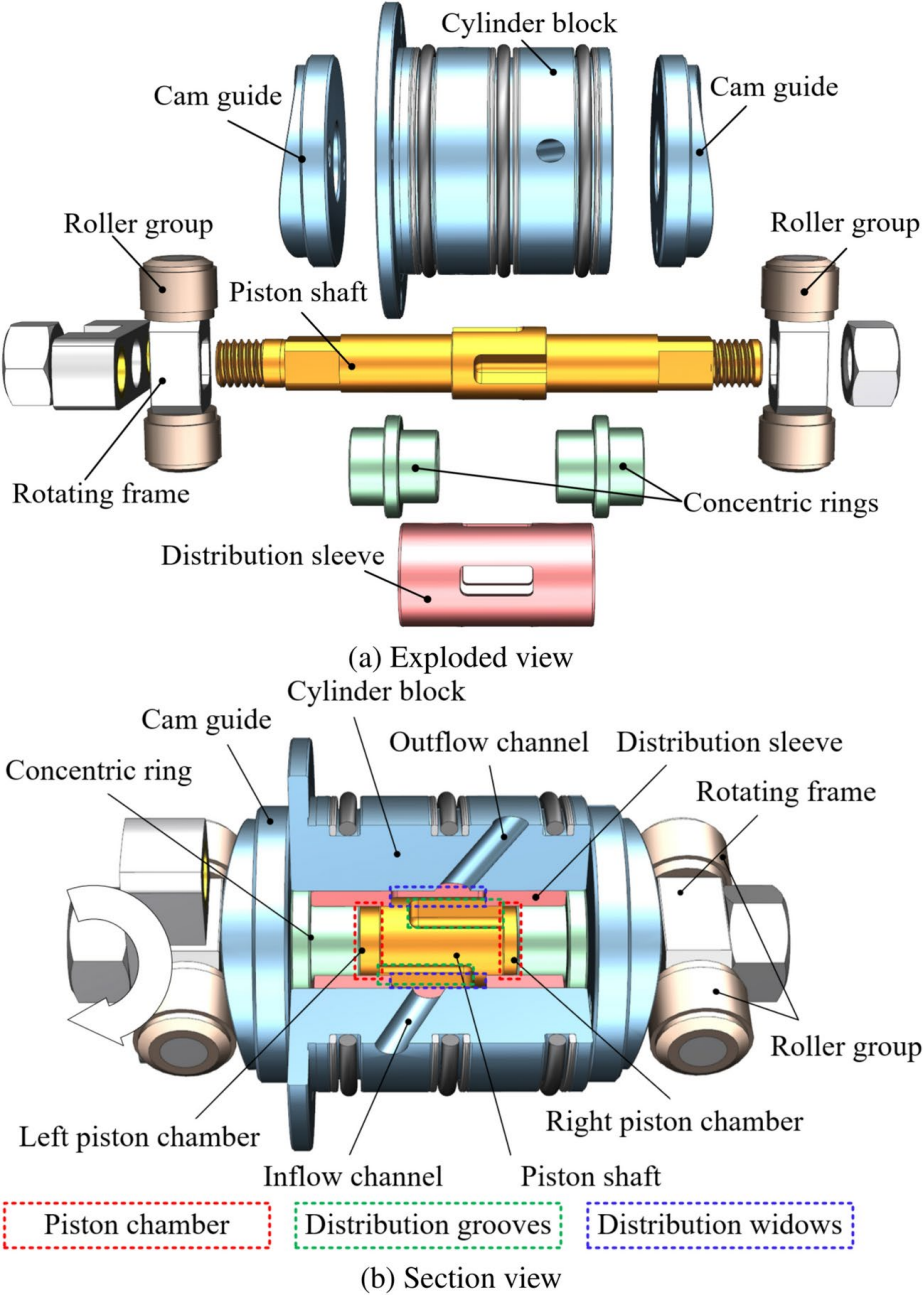
Structure of the cartridge 2D piston pump.

The cartridge-type 2D piston pump consists of two main components: the drive mechanism and the distribution mechanism, as shown in Fig. 1a. The drive mechanism includes a cam guide, roller assembly, rotating frame, and piston shaft. The roller assembly is divided into left and right sections, each comprising two cylindrical rollers and a rotating frame, with integrated bearings to minimize friction during movement. These rollers travel along an annular cam guide fixed to the cylinder body. This setup provides the necessary contact force for the rollers, enabling a combined rotational and axial reciprocating motion under the motor’s torque. This motion drives the piston shaft, leading to periodic changes in the internal chamber volume of the pump.

The piston shaft’s midsection features a shoulder, which, together with the concentric rings and distribution sleeve inside the cylinder body, creates a sealed dual-chamber structure, as shown in Fig. 1b. The distribution mechanism operates through distribution grooves on the piston shaft and a fixed distribution sleeve within the cylinder body, controlling the suction and discharge of hydraulic oil. The interference fit of the distribution sleeve with the cylinder block ensures effective sealing and stability. As the piston shaft rotates, the relative position of the distribution grooves and distribution windows shifts periodically, facilitating continuous and stable oil suction and discharge during pump operation.

### Pattern of axial motion

The integrated design of the piston shaft combines both reciprocating motion and distribution functions, thereby simplifying the structure of the separate distribution mechanism and reducing the number of components. The design of the cam guide and rollers eliminates the need for a complex oil film, achieving a simple and stable structure. This effectively enhances the overall efficiency and reliability of the pump.

The axial trajectory of the piston shaft in the 2D piston pump is designed as a sinusoidal function. Starting from a central position, the piston shaft performs reciprocating motion along the axial direction over time. The displacement and velocity as functions of time can be represented using sine and cosine functions:

1 $$\begin{aligned} s_a(t)= & \frac{h}{2} \sin (2\omega t) + \frac{h}{2} = \frac{h}{2} \sin (4 \pi n t) + \frac{h}{2} \end{aligned}$$

2 $$\begin{aligned} v_a(t)= & \frac{h}{2} \omega \cos (2\omega t) = \pi h n \cos (2 \pi n t) \end{aligned}$$

where $s_a$ is the axial displacement, $v_a$ is the axial velocity, $t$ is time, $h$ is the stroke of the cam guides, $\omega$ is the angular velocity around the axis of the piston shaft, $\omega = 2 \pi n$.

As the angular displacement of the piston shaft varies from 0 to 360°, the axial displacement undergoes two peaks and two troughs. This axial reciprocating motion causes periodic changes in the volumes of the left and right piston chambers, allowing the pump to complete four cycles of suction and discharge. The volume changes in the left and right piston chambers are directly correlated with the axial displacement of the piston shaft.

The oil discharge process in the left piston chamber is analyzed below, noting that the process in the right piston chamber is similar but with a 90° phase shift. The volume changes in the left piston chamber can be described by the following mathematical relationships:

3 $$\begin{aligned} V_L = V_d + A_p s_a \end{aligned}$$

where $V_d$ is the trapped volume of the piston chamber, and $V_L$ is the volume of the left piston chamber. In the design of hydraulic pumps, the actual work on the hydraulic oil is performed by the shoulder section of the piston, which is considered the effective working area $A_p$, represented as:

4 $$\begin{aligned} A_p = \frac{\pi }{4} (D_p^2 - d_p^2) \end{aligned}$$

where $D_p$ is the major diameter of the piston, and $d_p$ is the minor diameter of the piston.

### Pattern of flow area changes

**Fig. 2:**
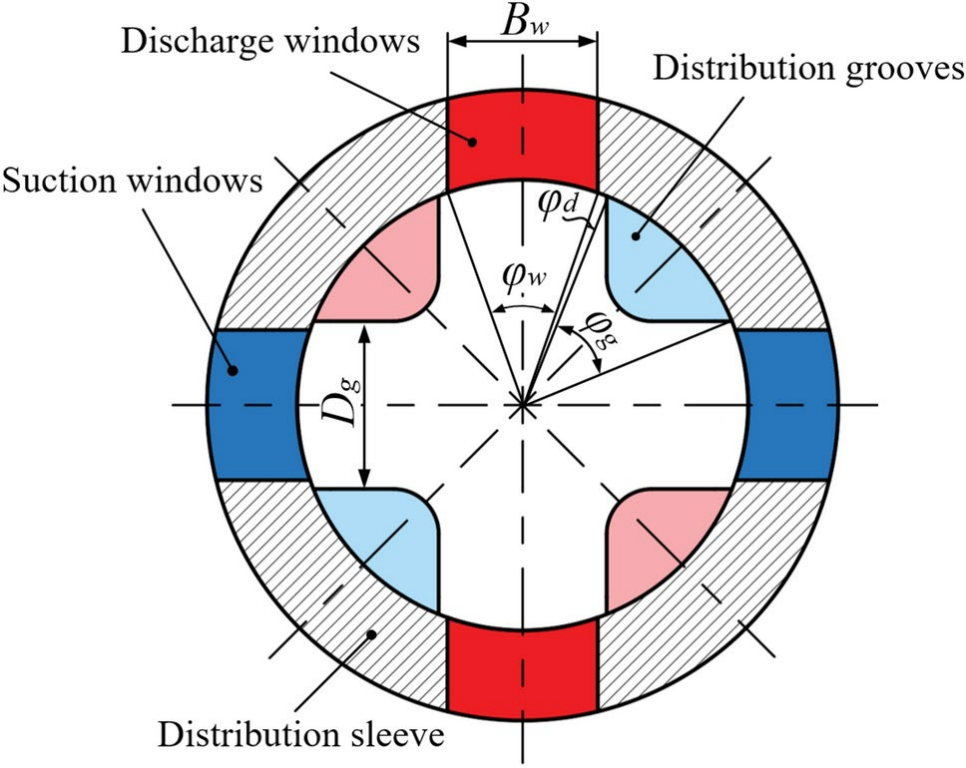
Sectional view of the piston chambers and distribution windows.

Figure 2 presents a cross-sectional view of the distribution windows and grooves. The distribution windows are machined by cutting a rectangular shape into the ring, with a width denoted as $B_w$. The distribution grooves are evenly spaced along the circumference, with the edges of adjacent grooves parallel to each other. The distance between them is $D_g$. The angles associated with the distribution windows, distribution grooves, and dead angles are represented by $\varphi _w$, $\varphi _g$, and $\varphi _d$, respectively, while the overlap angle is denoted by $\varphi _c$. These angles are defined as follows:

5 $$\begin{aligned} \varphi _w= & 2 \arcsin \left( \frac{B_w}{D_p} \right) \end{aligned}$$

6 $$\begin{aligned} \varphi _g= & \frac{\pi }{2} - 2 \arcsin \left( \frac{D_g}{D_p} \right) \end{aligned}$$

7 $$\begin{aligned} \varphi _d= & \frac{\pi }{4} - \frac{\varphi _w + \varphi _g}{2} \end{aligned}$$

8 $$\begin{aligned} \varphi _c= & \frac{\varphi _g - \varphi _w}{2} \end{aligned}$$

**Fig. 3:**
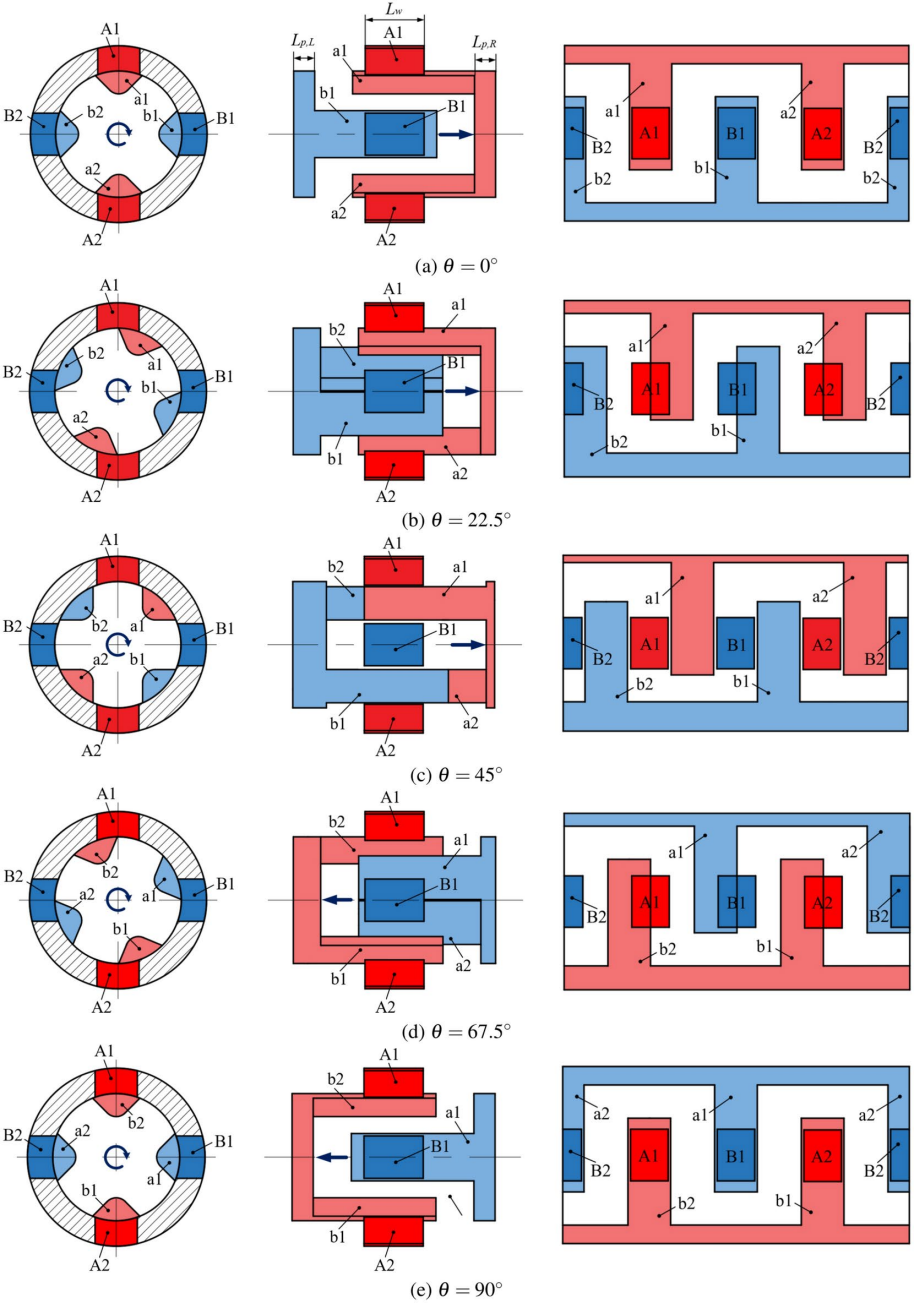
Suction and discharge principle of the 2D piston pump.

The arc length occupied by the distribution windows on the circumference is given by:

9 $$\begin{aligned} L_w = \varphi _w D_p \end{aligned}$$

where $L_w$ is the arc length occupied byy the distribution windows, as shown in Fig. 3a.

### Principle of suction and discharge

Figure 3 illustrates the process of a 2D piston pump completing one cycle of suction and discharge during a 90° rotation. In the figure, A1 and A2 represent the discharge windows, B1 and B2 represent the suction windows, while a1 and a2 denote the distribution grooves of the right piston chamber, and b1 and b2 denote the distribution grooves of the left piston chamber. The process is described in detail as follows:

- Initial position ($\theta = 0^\circ$): As shown in Fig. 3a, a1 and a2 are connected to A1 and A2 respectively, while b1 and b2 are connected to B1 and B2 respectively. At this position, the axial motion velocity and flow area are at their maximum. As the distribution grooves begin to rotate clockwise, the piston decelerates and moves to the right. At this stage, the left piston chamber enters the suction phase, while the right piston chamber is in the discharge phase.
- Rotation from *θ* = 0° to 45°: As shown in Fig. 3b, during this phase, the volume of the right piston chamber gradually decreases, with a1 connected to A1 and a2 connected to A2, maintaining the discharge state. Simultaneously, the volume of the left piston chamber increases, with b1 connected to B1 and b2 connected to B2, maintaining the suction state. As the piston continues to rotate towards 45°, it approaches the dead angle region $\theta \in \left[ \frac{\pi }{4} - \theta _d, \frac{\pi }{4} + \theta _d \right]$, as depicted in Fig. 3c. During this period, the piston chamber does not align with the distribution windows, resulting in a transitional phase where neither suction nor discharge occurs.
- Rotation from *θ* = 45° to 90°: As shown in Fig. 3c–e, after passing through the dead angle region, the leftward axial movement of the piston causes the volume of the left piston chamber to decrease, while the volume of the right piston chamber increases. Concurrently, as b1 and b2 in the left piston chamber connect with A2 and A1, the overlap area increases, facilitating the discharge process as the distribution windows adjust in coordination with the piston’s movement. For the right piston chamber, a1 and a2 begin to connect with B2 and B1, and the overlap area increases, initiating the suction process. During this stage, the functions of the left and right piston chambers interchange, ensuring the pump effectively circulates hydraulic fluid while continuously rotating. This process continues until the piston reaches the next dead angle region $\theta \in \left[ \frac{3\pi }{4} - \theta _d, \frac{3\pi }{4} + \theta _d \right],$ after which the functions switch again as the piston exits this region.

The flow area is determined by the product of the overlap width and the distribution window length, which can be calculated as follows:

10 $$\begin{aligned} S_{\text {out},L}(\theta )= & {\left\{ \begin{array}{ll} 0 & \theta \in \left[ 0, \frac{\pi }{4} - \varphi _d \right) \\ \frac{L_w D_p}{2} \left[ \theta - \left( \frac{\pi }{4} - \varphi _d \right) \right] & \theta \in \left[ \frac{\pi }{4} - \varphi _d, \frac{\pi }{4} + \varphi _d \right) \\ L_w B_w & \theta \in \left[ \frac{\pi }{4} + \varphi _d, \frac{\pi }{2} - \varphi _c \right) \\ L_w \left\{ B_w - \left[ \theta - \left( \frac{\pi }{2} - \varphi _c \right) \frac{D_p}{2} \right] \right\} & \theta \in \left[ \frac{\pi }{2} - \varphi _c, \frac{\pi }{2} + \varphi _c \right) \\ L_w B_w & \theta \in \left[ \frac{\pi }{2} + \varphi _c, \frac{\pi }{2} + \varphi _c + \varphi _w \right) \\ 0 & \theta \in \left[ \frac{\pi }{2} + \varphi _c + \varphi _w, \frac{5\pi }{4} - \varphi _d \right) \\ \frac{L_w D_p}{2} \left[ \theta - \left( \frac{5\pi }{4} - \varphi _d \right) \right] & \theta \in \left[ \frac{5\pi }{4} - \varphi _d, \frac{5\pi }{4} + \varphi _d \right) \\ L_w B_w & \theta \in \left[ \frac{5\pi }{4} + \varphi _d, \frac{3\pi }{2} - \varphi _c \right) \\ L_w \left\{ B_w - \left[ \theta - \left( \frac{3\pi }{2} - \varphi _c \right) \frac{D_p}{2} \right] \right\} & \theta \in \left[ \frac{3\pi }{2} - \varphi _c, \frac{3\pi }{2} + \varphi _c \right) \\ L_w B_w & \theta \in \left[ \frac{3\pi }{2} + \varphi _c, \frac{3\pi }{2} + \varphi _c + \varphi _w \right) \\ 0 & \theta \in \left[ \frac{3\pi }{2} + \varphi _c + \varphi _w, 2\pi \right] \end{array}\right. } \end{aligned}$$

11 $$\begin{aligned} S_{\text {in},L}(\theta )= & S_{\text {out},L}\left(\theta + \frac{\pi }{2}\right) \end{aligned}$$

where $S_{\text {out},L}$ represents the discharge flow area of the left piston chamber, and $S_{\text {in},L}$ denotes the suction flow area of the left piston chamber.

## Analysis and modeling

### Analysis of leakage losses

The axial flow caused by pressure differences and the tangential flow resulting from rotation are treated as independent phenomena, allowing for separate analysis of axial and circumferential flow mechanisms. Poiseuille flow, driven by a pressure difference, occurs in a slit between flat plates, where fluid velocity is highest at the center and decreases towards the walls. Couette flow, on the other hand, occurs between two parallel plates, with one plate moving relative to the other, generating a flow with velocity that varies linearly across the slit’s height.

**Fig. 4:**
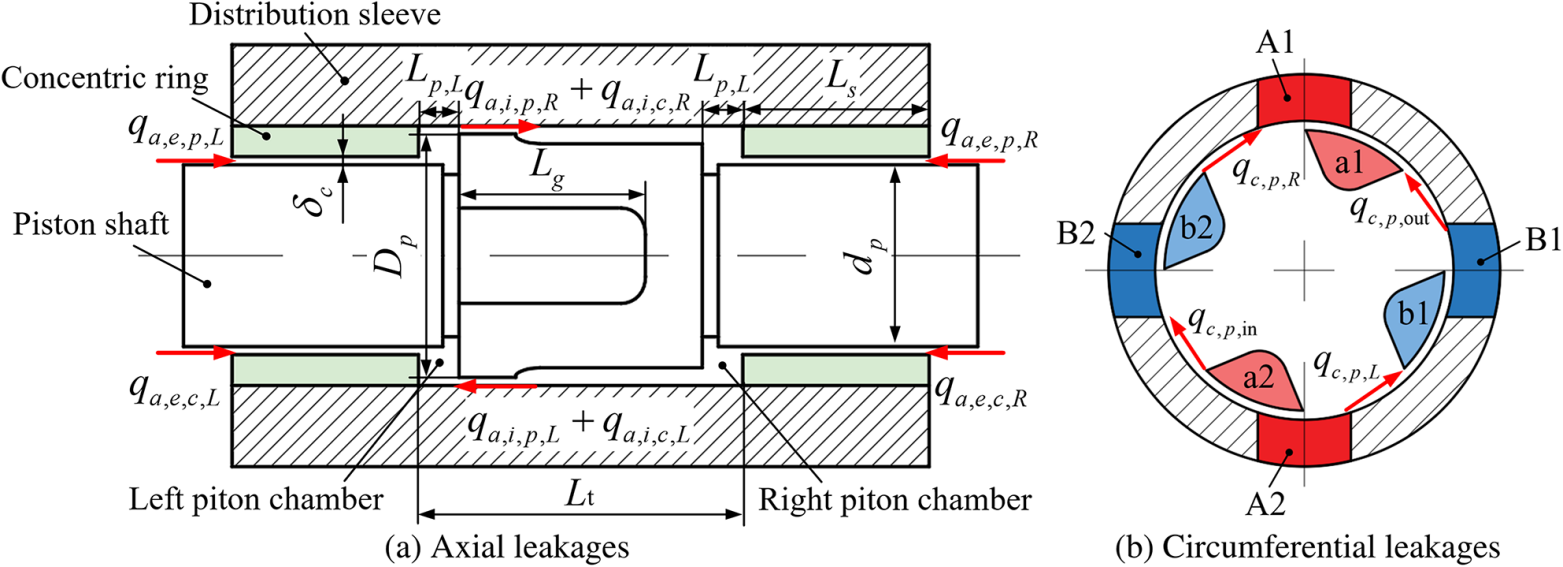
Leakage losses of the 2D piston pump.

In this analysis, flow into the chamber is considered positive. Leakage losses are categorized into two types: axial leakage and circumferential leakage. Axial leakage includes both internal and external leakage. As shown in Fig. 4a, internal leakage refers to oil leakage between the left and right piston chambers, while axial external leakage refers to oil leakage from the piston chamber to the outside, with the length of the sealing band remaining constant. Circumferential leakage, as depicted in Fig. 4b, occurs with the same slit width as axial leakage, but with a sealing band length that continuously varies.

The leakage flow rate is calculated using the geometric parameters of the leakage path and the pressure difference, applying the relevant flow equations. The following is the method for calculating the leakage flow rate.

#### Axial internal leakage

The Poiseuille flow rate for axial internal leakage is calculated as:

12 $$\begin{aligned} q_{a,i,p,L} = -\frac{\delta _c^3 (p_L - p_R)}{12 \mu _0} \left[ \frac{\pi D_p - 4B_g}{L_t - (L_{p,L} + L_{p,R})} + \frac{4B_g}{L_t - (L_{p,L} + L_{p,R}) - L_g} \right] \end{aligned}$$

where $\delta _c$ is the gap height; $\mu _0$ is the dynamic viscosity of hydraulic oil at normal temperature; $L_g$ is the length of the distribution groove; $L_{p,L}$ and $L_{p,R}$ are the lengths of the left and right annular piston chambers, respectively; $L_t$ is the total length of the piston chamber; $q_{a,i,p,L}$ is the Poiseuille flow rate of axial internal leakage for the left piston chamber; $p_R$ is the pressure in the right piston chamber; $p_L$ is the pressure in the left piston chamber; and $B_g$ is the arc length of the distribution groove on the circumference, expressed as:

13 $$\begin{aligned} B_g = \frac{\varphi _g D_p}{2} \end{aligned}$$

The Couette flow, also considered positive when flowing into the chamber, is given by:

14 $$\begin{aligned} q_{a,i,c,L} = -\frac{\pi D_p v_a \delta _c}{2} \end{aligned}$$

As a result, the total axial external leakage flow rate can be expressed as:

15 $$\begin{aligned} q_{a,i,L} = q_{a,i,p,L} + q_{a,i,c,L} \end{aligned}$$

#### Axial external leakage

The Poiseuille flow rate for axial external leakage is given by:

16 $$\begin{aligned} q_{a,e,p,L} = -\frac{\delta _c^3}{12 \mu _0} \cdot \frac{\pi d_p}{L_s} \cdot (p_L - p_{\text {in}}) \end{aligned}$$

where $L_s$ is the length of the concentric ring; $q_{a,e,p,L}$ is the Poiseuille flow rate for the axial external leakage of the left piston chamber.

The Couette flow, which is negative when leaking to the outside, is expressed as:

17 $$\begin{aligned} q_{a,e,c,L} = \frac{\pi d_p v_a \delta _c}{2} \end{aligned}$$

Therefore, the total axial external leakage flow rate is:

18 $$\begin{aligned} q_{a,e,L} = q_{a,e,p,L} + q_{a,e,c,L} \end{aligned}$$

#### Circumferential internal leakage

Circumferential Couette flow involves the exchange of flow between discharge and suction windows, which theoretically cancel each other out. Poiseuille flow, however, represents leakage from the discharge chamber and windows to the suction chamber and windows.

As the pump rotates from 0° to 90°, the gap length decreases, stabilizes, and then decreases again. The gap width is expressed as:

19 $$\begin{aligned} B_c = {\left\{ \begin{array}{ll} \left( \frac{\pi }{2} - \varphi _g \right) \frac{D_p}{2} & \theta \in \left[ 0, \varphi _c\right) \\ \left[ \left( \frac{\pi }{2} - \varphi _g \right) - \left( \theta - \varphi _c \right) \right] \frac{D_p}{2} & \theta \in \left[ \varphi _c, \varphi _c + \varphi _w + 2\varphi _d \right) \\ \left[ 2\varphi _d + \theta - \left( \varphi _c + \varphi _w + 2\varphi _d \right) \right] \frac{D_p}{2} & \theta \in \left[ \varphi _c + \varphi _w + 2\varphi _d, \frac{\pi }{2} - \varphi _c \right) \\ \left( \frac{\pi }{2} - \varphi _g \right) \frac{D_p}{2} & \theta \in \left[ \frac{\pi }{2} - \varphi _c, \frac{\pi }{2} \right] \end{array}\right. } \end{aligned}$$

According to the Poiseuille’s equation, as the gap width $B_c$ approaches zero just before communication, the flow rate theoretically becomes infinite, which is not realistic. Instead, the flow through the gap is calculated using the orifice equation:

20 $$\begin{aligned} q_0 = C_d A_0 \sqrt{\frac{2 \Delta p}{\rho }} \end{aligned}$$

where $A_0$ represents the flow area just before the right piston chamber connects to the suction windows:

21 $$\begin{aligned} A_0 = \delta _c L_w \end{aligned}$$

Based on this approach and the Poiseuille’s equation, with inflow considered positive, and similar leakage occurring at two locations, the leakage flow rates at the discharge windows, suction windows, right piston chamber, and left piston chamber are given by:

22 $$\begin{aligned} q_{c,p,\text {out}}= & \text {sign}(q_1) \cdot \min \{ |q_1|, q_0 \}, \quad q_1 = -\frac{2 \delta _c^3 L_w}{12 \mu _0 B_c} \left[ (p_{\text {out}} - p_R) + (p_{\text {out}} - p_L) \right] \end{aligned}$$

23 $$\begin{aligned} q_{c,p,L}= & \text {sign}(q_2) \cdot \min \{ |q_2|, q_0 \}, \quad q_2 = -\frac{2 \delta _c^3 L_w}{12 \mu _0 B_c} \left[ (p_L - p_R) + (p_L - p_{\text {in}}) \right] \end{aligned}$$

where $q_1$ and $q_2$ are temporary flow rate variables.

### Establishment of flow characteristic equation

#### Reynolds number

The above methods for calculating gap flow using Poiseuille’s and Couette’s equations are based on the assumption of laminar flow. This assumption can be validated by determining the flow regime using the Reynolds number, which is defined as:

24 $$\begin{aligned} \text {Re} = \frac{\rho _0 U d_h}{\mu _0} \end{aligned}$$

where $U$ is the flow velocity, and $d_h$ is the hydraulic diameter. For the annular gaps in axial internal and external leakage:

25 $$\begin{aligned} d_h = \frac{4 \delta _c}{\pi } \end{aligned}$$

For circumferential leakage:

26 $$\begin{aligned} d_h = 2 \delta _c \end{aligned}$$

The Reynolds number at 3000 rpm and 35 MPa serves as a validation benchmark, as these conditions result in the highest values. The preliminary calculations, obtained through AMESIM simulation, show that the Reynolds numbers are approximately 0.230 for axial external leakage, 0.322 for axial internal leakage, and 309 for circumferential internal leakage. The critical Reynolds number ($Re_{\text {crit}}$) for the transition from laminar to turbulent flow is 1100^28^, indicating that the leakage flow model based on these values is valid for laminar flow.

However, during the discharge process, the Reynolds number reaches approximately 3446 at the moment of reverse flow, which exceeds the critical threshold, indicating turbulent flow. Therefore, the flow rate during this phase must be determined using Eq. (31).

#### Bulk modulus of hydraulic oil

The compressibility of hydraulic oil impacts volumetric efficiency primarily because, under high pressure, the volume of compressible oil decreases. This means that even though the mechanical components of the pump may ideally provide a constant flow output, the actual amount of oil discharged will be less than the theoretical value due to oil compression. Consequently, as the system pressure increases, the pump’s volumetric efficiency decreases. Therefore, it is essential to consider the effects of the oil’s compressibility, viscosity, air content, and flow state.

In this chapter, the IFAS model^29^ is used to calculate the effective bulk modulus of oil containing gas. This model has been experimentally validated by Kim et al.^30^. The effective bulk modulus from the IFAS model is given by:

27 $$\begin{aligned} K_{\text {ef}} = \frac{(1 - \alpha ) \left[ 1 + \frac{m_p (p - p_0)}{E_0} \right] ^{-{\frac{1}{m_p}}} + \alpha \left( \frac{p_0}{p} \right) ^{\frac{1}{\kappa }}}{\frac{1}{E_0} (1 - \alpha ) \left[ 1 + \frac{m_p (p - p_0)}{E_0} \right] ^{-{\frac{m_p + 1}{m_p}}} + \frac{\alpha }{\kappa p_0} \left( \frac{p_0}{p} \right) ^{\frac{\kappa + 1}{\kappa }}} \end{aligned}$$

where $\alpha$ is the gas volume fraction; $K_{\text {ef}}$ is the effective bulk modulus of oil containing gas; $\rho _0$ is the oil density at atmospheric pressure; $p_0$ is the standard atmospheric pressure, taken as 101,325 Pa; $p$ is the absolute pressure; $\kappa$ is the polytropic index of air, taken as 1.15; $m_p$ is the pressure-related parameter in the oil’s bulk modulus, taken as 11.4^30^; and $E_0$ is the bulk modulus of pure hydraulic oil.

#### Flow coefficient

The flow coefficient $C_d$ in Eq. (20) is a parameter that describes the efficiency of fluid flow through throttling elements (such as valves, nozzles, or orifices). It is influenced by geometric shape, flow state (laminar or turbulent), Reynolds number, operating pressure and temperature, physical properties of the fluid (such as viscosity and density), and inlet and outlet flow conditions.

Introducing the flow number, defined as:

28 $$\begin{aligned} \lambda = \frac{d_h \rho _0}{\mu _0} \sqrt{\frac{2 |\Delta p|}{\rho _0}} \end{aligned}$$

The flow coefficient is given by:

29 $$\begin{aligned} C_d = C_{d,\text {max}} \cdot \tanh \left( \frac{2 \lambda }{\lambda _{\text {crit}}} \right) \end{aligned}$$

where $C_{d,\text {max}}$ is the maximum flow coefficient, typically 0.7; $\lambda _{\text {crit}}$ is the critical flow number for the transition from laminar to turbulent flow, given by:

30 $$\begin{aligned} \lambda _{\text {crit}} = \frac{\text {Re}_{\text {crit}}}{C_{d,\text {max}}} \end{aligned}$$

#### Flow characteristic equation

When considering the piston chamber as a closed volume, it is necessary to account for both the volume changes caused by the reciprocating motion of the piston and the mass changes due to fluid exchange between the distribution windows and the piston chamber. Therefore, the relationship between the pressure and volume changes within the piston chamber can be described by the following differential expression:

31 $$\begin{aligned} \frac{dp_L}{dt} = \frac{K_{\text {ef}}}{V_L} \left( -\frac{dV_L}{dt} + q_{a,i,L} + q_{a,e,L} + q_{c,p,L} - q_{\text {out},L} \right) \end{aligned}$$

where $q_{\text {out},L}$ is the instantaneous flow rate of oil through the discharge windows from the left piston chamber:

32 $$\begin{aligned} q_{\text {out},L} = C_{d,\text {out},L} S_{\text {out},L} \sqrt{\frac{2 |p_L - p_{\text {out}}| }{\rho }} \cdot \frac{\rho }{\rho _0} \cdot \text {sign}(p_L - p_{\text {out}}) \end{aligned}$$

The hydraulic oil ultimately flows out of the pump from the discharge chamber. Since the volume of the discharge chamber remains constant, the flow characteristics can be described by the following equation:

33 $$\begin{aligned} \frac{dp_{\text {out}}}{dt} = \frac{K_{\text {ef}}}{V_{\text {out}}} \left( q_{\text {out},L} + q_{\text {out},R} + q_{c,p,\text {out}} - q_{\text {out}} \right) \end{aligned}$$

where $p_{\text {out}}$ is the pressure in the discharge chamber, $V_{\text {out}}$ is the volume of the discharge chamber, and $q_{\text {out}}$ is the dynamic output flow rate of the pump.

Equations (31) and (33) encompass the influences of leakage losses, flow state, oil compressibility, and flow coefficients. By solving these three equations, the key state parameters such as pressure, output flow rate, and leakage flow rate within the left and right piston chambers and the discharge chamber can be determined. The final volumetric efficiency calculation result can be obtained as follows:

34 $$\begin{aligned} \eta _{v,\text {th}} = \frac{\int _{t_0}^{t_0+T} q_{\text {out}} \, dt}{V_g} \end{aligned}$$

where $\eta _{v,\text {th}}$ is the theoretical volumetric efficiency, $t_0$ is the initial time of an arbitrary cycle, and $T$ is the period of one complete pump rotation, $T = 2\pi /\omega$.

## Analysis and discussion

### Parameter settings

As shown in Table 1, the structural parameters of the cartridge 2D piston pump are detailed. These dimensions correspond to the annotations in Figs. 2, 3 and 4.

**Table 1:** Geometrical parameters of the cartridge 2D piston pump (0.76 mL/r).

Symbol	Description	Value
$L_t$	Piston chamber total length	24 mm
$L_g$	Distribution groove length	14.5 mm
$D_g$	Distribution groove interval	5.8 mm
$D_p$	Piston chamber major diameter	15 mm
$d_p$	Piston chamber minor diameter	12 mm
$\theta _0$	Distribution groove width angle	$\pi /2$
$B_w$	Distribution window width	5 mm
$L_w$	Distribution window length	7 mm
$L_{p,L}, L_{p,R}$	Circumferential chamber length	1.5–4.5 mm
*h*	Cam guide stroke	3 mm
$\delta _c$	Gap height	5 μm

ISO VG 15 hydraulic oil was used as the working medium, which is commonly applied in the fields of robotics and aerospace. Since the oil operates in an open-loop system during the experiment and the pump is continuously cooled by a large amount of hydraulic oil, the experimental conditions are assumed to be isothermal. At room temperature (25 °C), its dynamic viscosity is 0.01732 Pa.s, and its density is 837 kg/m$^3$. The air content in the oil significantly affects its bulk modulus, thus impacting the calculation results. At room temperature and atmospheric pressure, the air content in the hydraulic oil is 6%.

**Fig. 5:**
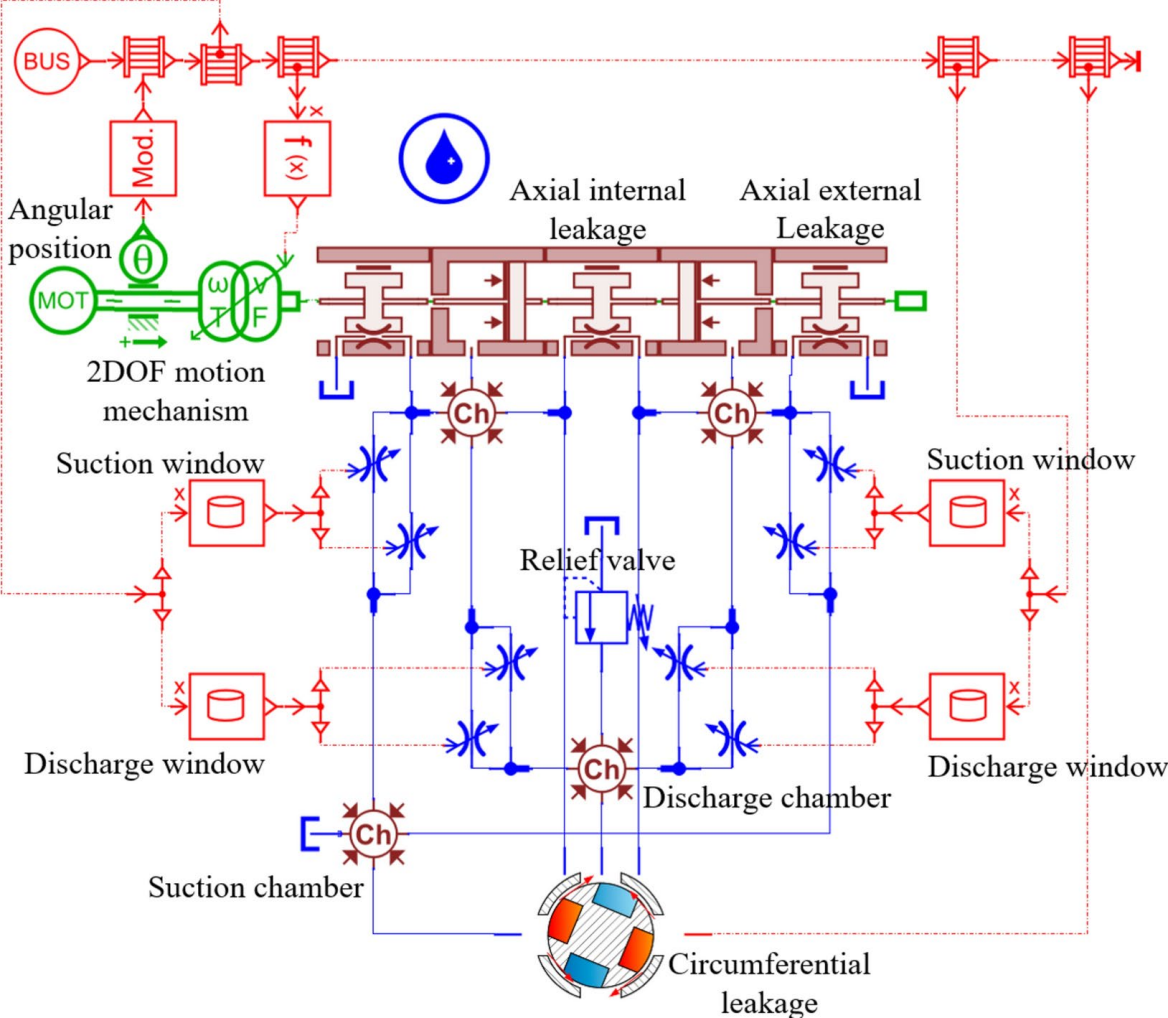
Co-simulation model of AMESim and Simulink.

To facilitate calculation and analysis, AMESim and Simulink were used for co-simulation. Details can be found in Fig. 5. The variable-step ode45 solver was chosen to optimize computational efficiency while ensuring high accuracy (relative error reaching $10^{-7}$).

### Simulation results

#### Reverse flow loss

**Fig. 6:**
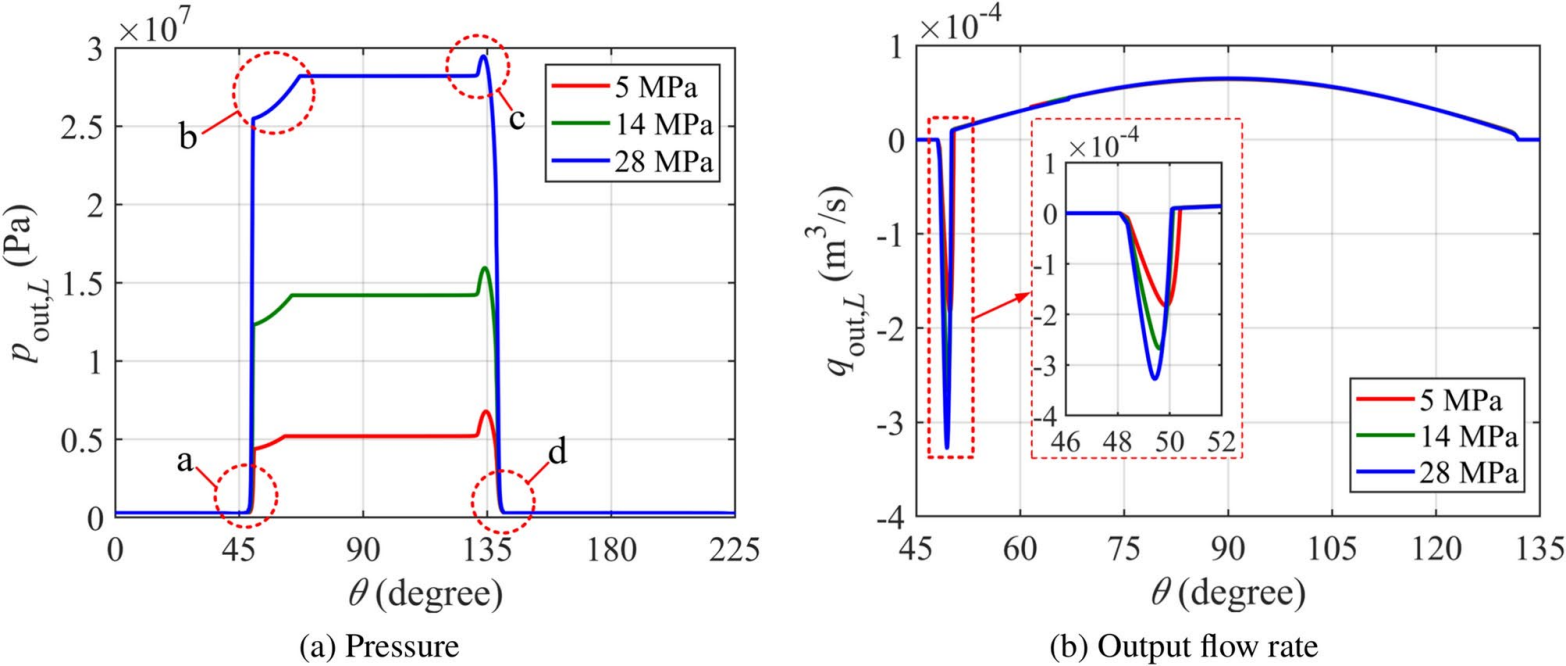
Pressure and output flow rate of the left piston chamber at different pressure.

In Fig. 6a, the pressure variation in the left piston chamber during one discharge cycle is illustrated. Between 0 and 45°, the left piston chamber is in a suction state. As the rotational angle surpasses 45° and reaches point a, the pressure rapidly increases. At this point, the left piston chamber establishes a direct connection with the discharge window, allowing high-pressure oil to reverse flow into the chamber due to the pressure difference.

This reverse flow phenomenon is further demonstrated in Fig. 6b, where it is shown that the volume of reverse flow is positively correlated with the pressure difference. The reverse flow reduces the oil volume in the discharge chamber, causing a slight pressure drop below the preset outlet pressure. As a result, at region b, the discharge chamber pressure has not yet recovered to the set outlet pressure, creating a local dip in the pressure curve.

In region c, the piston reaches the dead angle $\theta _d$. As the piston continues moving left and compressing the trapped oil, a pressure peak forms due to the closed distribution window and the confined oil. Finally, in region d, as the piston chamber connects with the suction window, the pressure rapidly drops to the inlet pressure.

When comparing the pressure and flow curves for three different pressure levels, it is observed that as the pressure increases, the peak value of reverse flow also rises. However, the angle over which the reverse flow occurs remains almost unchanged across the different pressure levels.

**Fig. 7:**
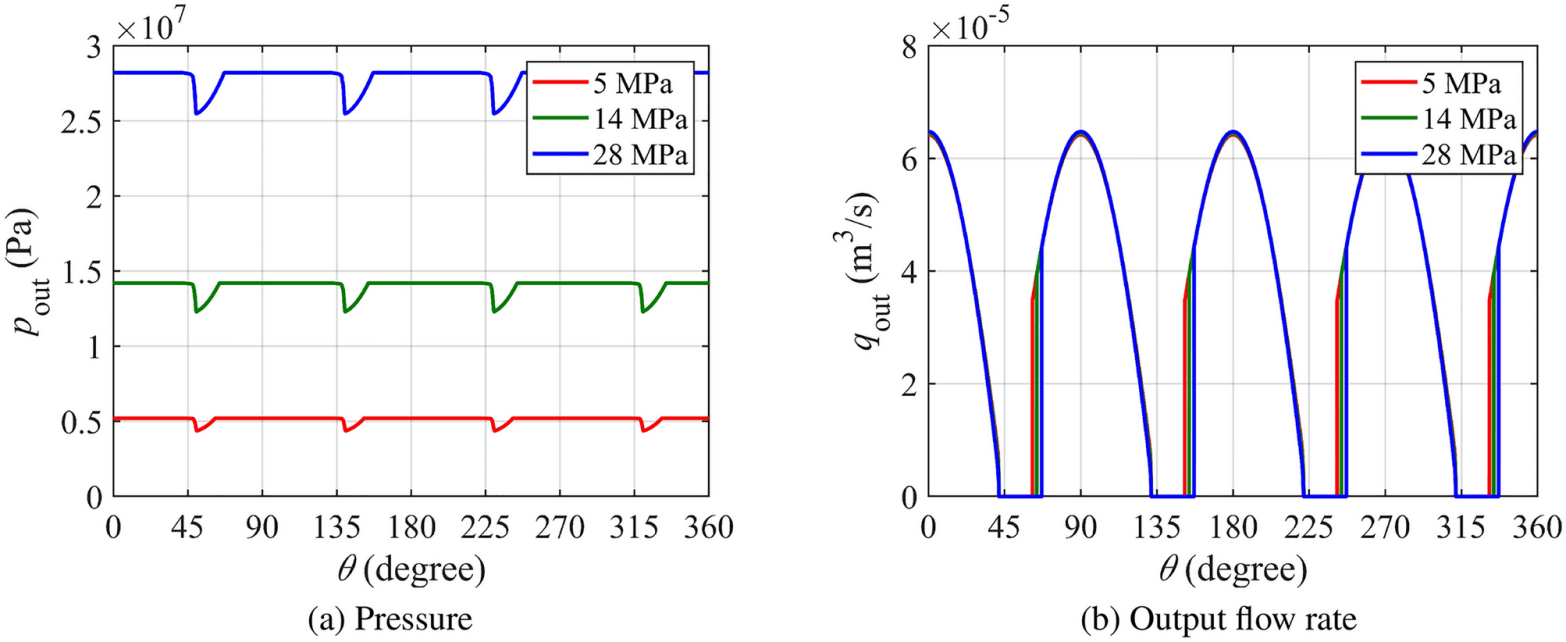
Pressure and output flow curves of the oil discharge chamber at different pressure.

Figure 7a shows the pressure fluctuations in the discharge chamber, where dips occur near 45°, 135°, 225°, and 15° due to oil reverse flow. The subsequent pressure rise is caused by oil replenishment in the piston chamber. As seen in Fig. 7b, reverse flow reduces the output flow rate by preventing oil delivery until the pressure recovers to the load pressure, causing a sharper drop in discharge pressure and shortening the duration of high-pressure oil discharge, thereby reducing volumetric efficiency.

#### Leakage losses

**Fig. 8:**
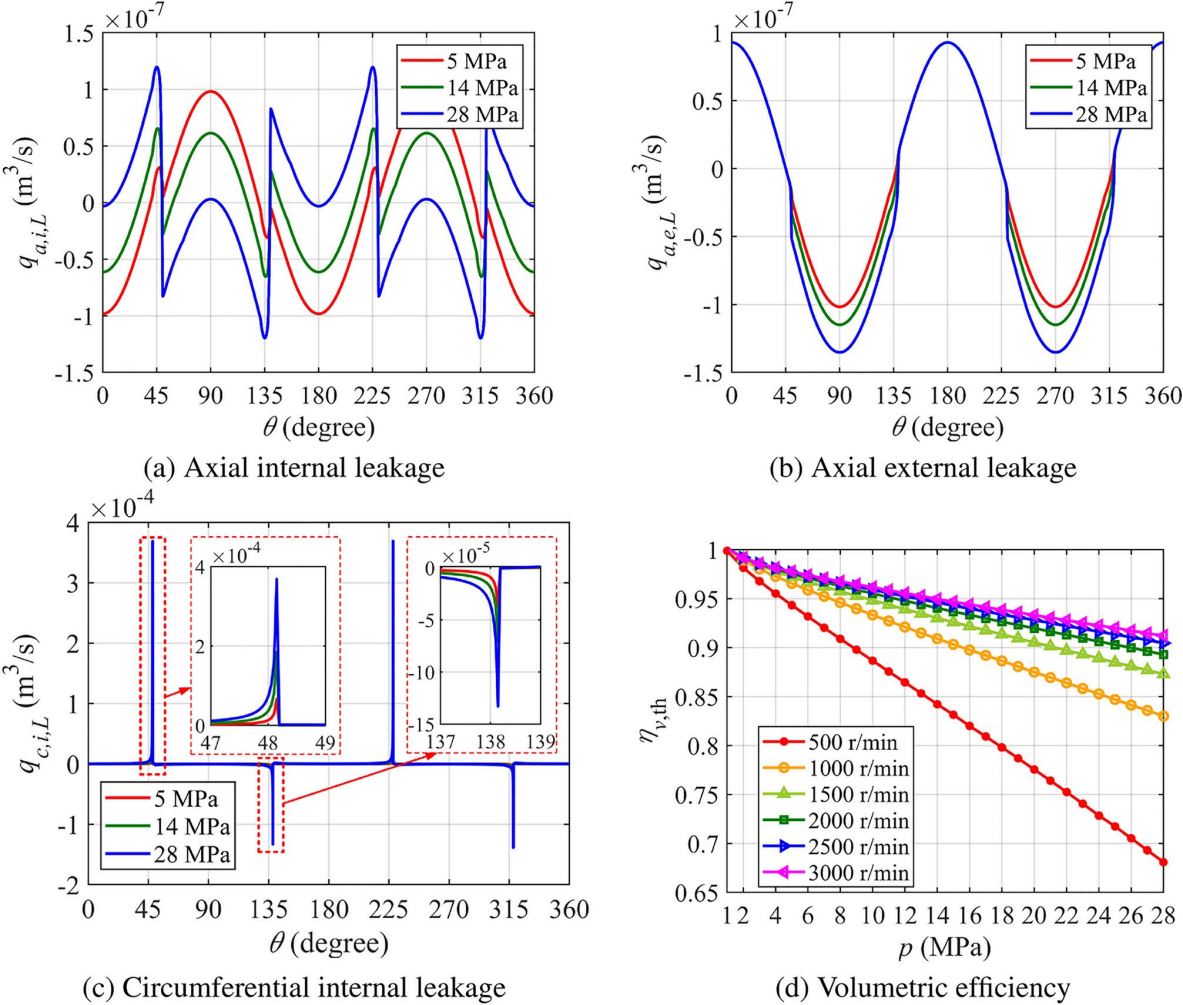
Leakage flow rate curves of the left piston chamber and volumetric efficiency of the pump.

Figure 8a shows the axial internal leakage flow rate between the left and right piston chambers. From 0° to around 45°, the left piston chamber is in the suction phase as the piston moves to the right, meaning axial internal leakage has minimal impact on volumetric efficiency. However, after 45°, as the piston moves left and pressure increases in the left chamber, Poiseuille flow, driven by the pressure difference, pushes oil from the left chamber to the right, while Couette flow, driven by piston movement, flows in the opposite direction. The combination of these flows shapes the curve between 45° and 135°. Poiseuille flow contributes to leakage losses, reducing volumetric efficiency by diverting oil away from the desired output, whereas Couette flow mitigates some of this loss. Despite their opposing directions, both flows are part of the leakage losses. At lower pressures, Couette flow plays a more dominant role and helps improve efficiency, but at higher pressures, Poiseuille flow takes over, resulting in net negative flow and efficiency losses.

Figure 8b depicts the axial external leakage throughout the cycle. Between 45° and 135°, both Poiseuille and Couette flows contribute to leakage, reducing volumetric efficiency. As pressure increases, so does leakage, further diminishing efficiency.

Figure 8c illustrates circumferential leakage over one cycle. A peak near 45° is caused by high-pressure oil reverse flow into the left chamber, and another peak near 135° occurs when high pressure causes oil to leak into the suction chamber. Both of these peaks contribute to volumetric efficiency losses, and as pressure rises, the peaks grow larger, resulting in greater losses.

Figure 8d highlights how volumetric efficiency changes with speed and pressure. As speed increases, efficiency improves because the total theoretical flow increases faster than the rise in leakage and reverse flow, reducing their impact proportionally. Speed significantly affects both leakage and reverse flow, playing a key role in overall performance.

Conversely, when pressure increases at a constant speed, volumetric efficiency decreases. Higher pressure amplifies both leakage and reverse flow, resulting in more flow being lost and reduced efficiency. This makes pressure a major factor driving these efficiency losses.

#### Minimize trapped volumes

From the above analysis, it can be seen that at 3000 r/min and 28 MPa, axial leakage, circumferential leakage, and reverse flow account for 0.44%, 43.35%, and 56.21%, respectively. Reducing the gap size has little effect on improving volumetric efficiency, while minimizing circumferential leakage and reverse flow is the most effective approach.

**Fig. 9:**
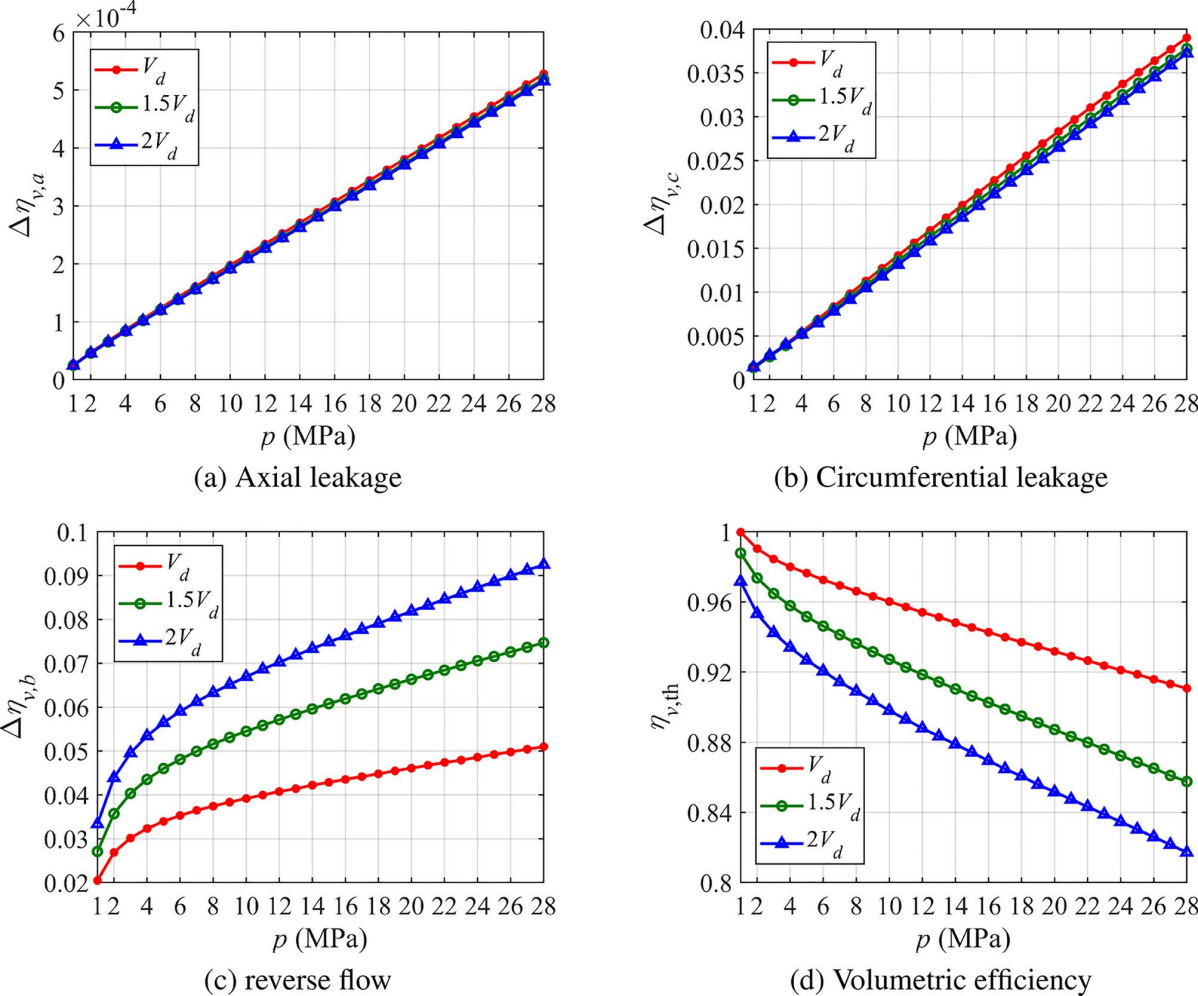
Volumetric efficiency and losses with different trapped volume sizes.

Figure 9 shows how the magnitude of leakage and reverse flow varies under three different trapped volume conditions: $V_{d}$, 1.5$V_{d}$, and 2$V_{d}$. From Fig. 9a,b, it can be seen that both axial leakage and circumferential leakage increase linearly with pressure, and the three lines corresponding to different trapped volumes are very close to each other. However, Fig. 9c reveals that reverse flow loss increases sharply at first with rising pressure and then transitions to a near-linear growth. The curves clearly show that as the trapped volume decreases, reverse flow loss becomes smaller. As shown in Fig. 9d, volumetric efficiency increases significantly as the trapped volume decreases, reaching 81.72%, 86.76%, and 91.22% at 3000 r/min and 28 MPa for the conditions of $V_{d}$, 1.5$V_{d}$, and 2$V_{d}$, respectively.

## Experimental verification

### Test rig description

**Fig. 10:**
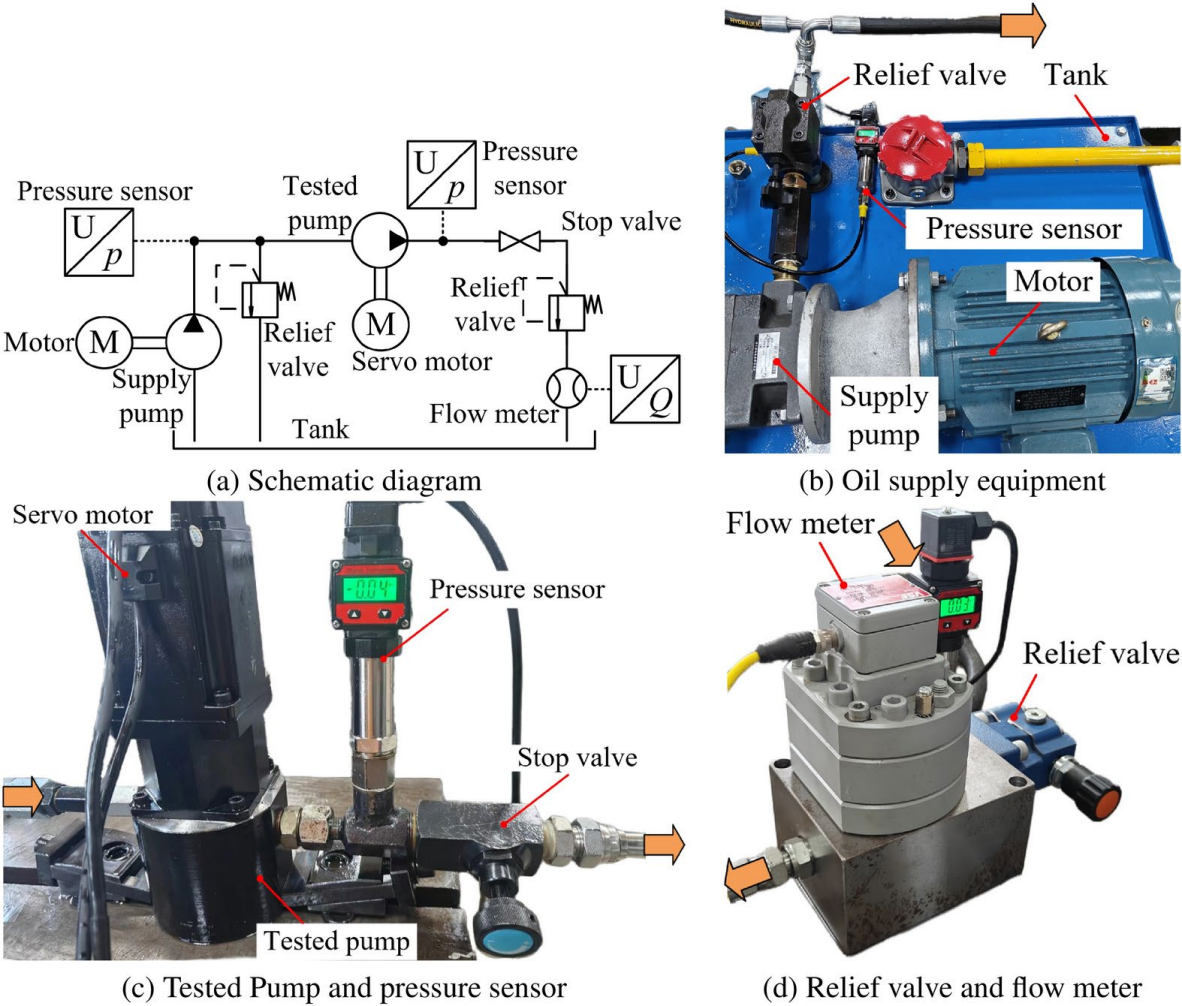
Volumetric efficiency test rig.

**Fig. 11:**
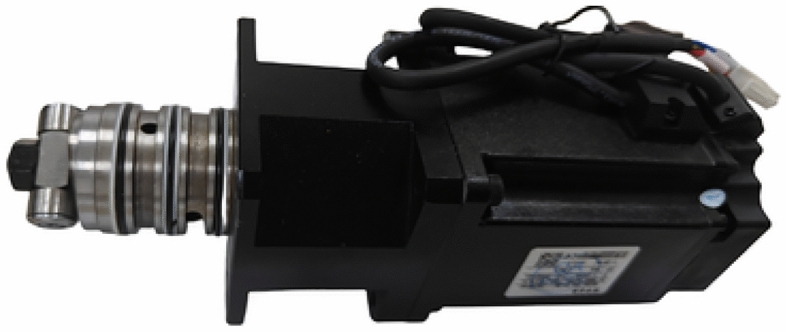
Tested motor-driven 2D piston pump.

As shown in Fig. 10, the volumetric efficiency test bench for the motor-driven 2D piston pump is used. ISO VG 15 aviation hydraulic oil is supplied to the pump under test, and a pressure sensor is installed at the outlet of the supply pump (Fig. 11). The supply pressure is controlled by adjusting the relief valve, with a maximum possible pressure of 1 MPa. However, excessively high pressure may induce unnecessary vibrations in the pump under test. To avoid this and ensure proper oil suction, the supply pressure is maintained at 0.3 MPa during the experiment.

The pump under test is driven by a servo motor, with its rotational speed precisely controlled by a dedicated controller. A throttle valve is installed at the outlet of the pump under test, and it is adjusted based on the readings from a pressure sensor before the throttle valve to set the outlet pressure. A flow meter is installed at the end of the throttle valve to measure the outlet flow, and a safety valve is positioned nearby to prevent excessive pressure in the oil circuit. Figure 10 is the photograph of tested pump.

In the above test rig, the parameters and specifications of the outlet pressure sensor and flow meter of the pump under test affect the accuracy of the experimental results. Detailed information is listed in Table 2.

**Table 2:** Details of related sensors of the test rig.

Name	Type	Parameters
Pressure sensor	QDW90A	Range: 0–315 bar; accuracy: ± 0.2%
Flow meter	VSE-58809	Range: 0.05–80 L/min; accuracy: ± 0.3%

### Process and results

Set the inlet pressure by regulating the relief valve on the supply pump, using the pressure sensor for reference. Start the servo motor and set the pump speed within the range of 500-3000 r/min. Adjust the throttle valve at the outlet to maintain an outlet pressure between 1-28 MPa, based on the pressure sensor readings. Analyze the experimental data and calculate the final volumetric efficiency according to its definition:

35 $$\begin{aligned} \eta _{v,\text {test}} = \frac{q_{\text {out,test}}}{V_g \cdot n} \end{aligned}$$

where $q_{{\text {out,test}}}$ represents the tested output flow rate, while $\eta _{v,\text {test}}$ denotes the tested volumetric efficiency.

**Fig. 12:**
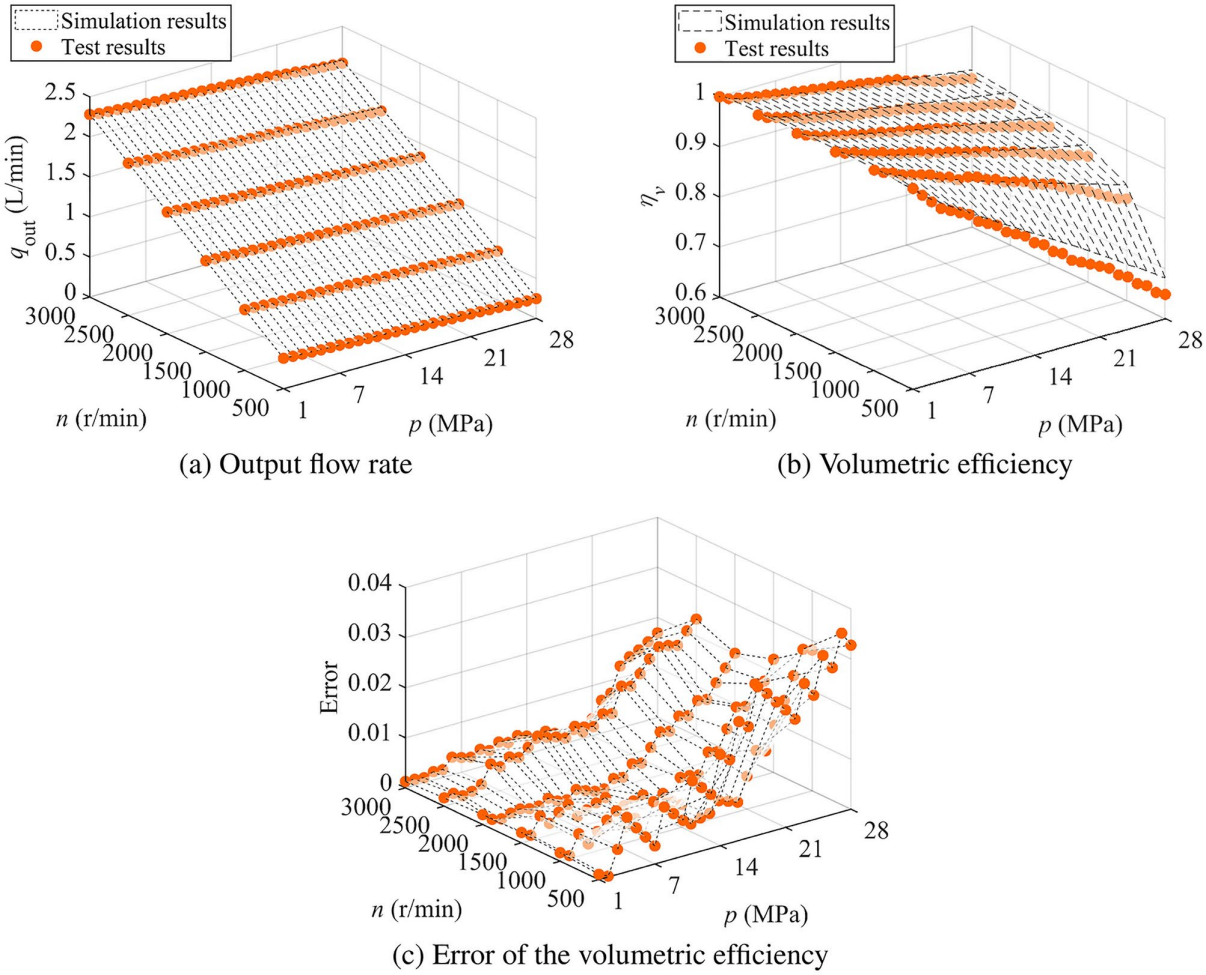
Comparison of simulation and test results of output flow rate and volumetric efficiency of the pump.

The experimental data, shown in Fig. 12a,b, compare the flow rate and volumetric efficiency obtained from both simulation and experimentation, while Fig. 12c highlights the errors between the two under various operating conditions. The results show that volumetric efficiency increases with higher rotational speeds and decreases as pressure rises. At 500 r/min and 28 MPa, the lowest tested efficiency was 64.81%, slightly below the simulated value of 68.09%, resulting in a deviation of 3.28%, the largest observed discrepancy. At 3000 r/min, efficiency followed a declining trend with increasing pressure, dropping to 89.53% at 28 MPa, compared to the simulated result of 91.22%, with a deviation of 1.69%.

The maximum error of 3.57% occurred at 500 r/min and 28 MPa. The trend suggests that the discrepancy increases with higher pressures, indicating that the discharge window flow coefficient $C_d$ during the reverse flow phase may require further adjustment to a larger value. Additional experimental data are needed to validate this refinement.

Nevertheless, the experimental results remain highly reliable. The strong correlation between the experimental and simulated data confirms the model’s accuracy and effectiveness in predicting volumetric efficiency across a speed range of 500–3000 r/min and a pressure range of 1–28 MPa.

## Conclusions

This paper presents a volumetric efficiency model for high-pressure motor-driven 2D piston pumps, accounting for key factors such as axial internal leakage, axial external leakage, circumferential leakage, and reverse flow. To improve the model’s accuracy, additional elements like hydraulic fluid compressibility, turbulence, and flow coefficients were considered. The model was developed using a co-simulation environment integrating AMESim and Simulink, and its accuracy was validated through experimental testing.

The experimental results confirmed that rotational speed increases volumetric efficiency, as it has little impact on leakage and reverse flow. On the other hand, pressure plays a significant role in reducing efficiency due to its greater influence on leakage and reverse flow. The study found that axial leakage contributes relatively little to overall losses, while circumferential leakage and reverse flow are the dominant factors. Reducing trapped volume was identified as an effective way to minimize reverse flow, which could improve efficiency.

Looking ahead, further refinements to the flow coefficient $C_d$ using additional experimental data could enhance the model’s precision. Moreover, other strategies to introduce high-pressure oil into the piston chamber before the discharge phase, aimed at reducing reverse flow and improving volumetric efficiency, include incorporating damping grooves and utilizing PCFV.

## Data Availability

All data needed to evaluate the conclusions in the paper are present in the paper and/or the Supplementary Materials. Additional data related to this paper may be requested from the corresponding author.

## List of symbols

$A_0$: Flow area at the moment just before the right piston chamber connects to the suction windows ($\hbox {m}^2$)
$A_p$: Effective working area of the piston ($\hbox {m}^2$)
$$B_c$$: Gap width (m)
$B_g$: Arc length of the distribution groove on the circumference (m)
$B_w$: Width of the distribution windows (m)
$$C_d$$: Flow coefficient
$C_{d,\text {max}}$: Maximum flow coefficient, typically 0.7
$$C_w$$: Wetted perimeter (m)
$D_g$: Distribution groove distance (m)
$D_p$: Piston chamber major diameter (m)
$$d_h$$: Hydraulic diameter (m)
$d_p$: Piston chamber minor diameter (m)
$E_0$: Bulk modulus of pure hydraulic oil (Pa)
$h$: Cam guide stroke (m)
$K_\text {ef}$: Effective bulk modulus of oil containing gas (Pa)
$L_g$: Piston chamber total length (m)
$L_{p,L}$: Length of the left annular piston chambers (m)
$L_{p,R}$: Length of the right annular piston chambers (m)
$L_s$: Length of the concentric ring (m)
$L_t$: Piston chamber total length (m)
$L_w$: Arc length occupied by the distribution windows (m)
$m_p$: Pressure-related parameter in the oil’s bulk modulus, taken as 11.4
$n$: Input rotational speed (r/min)
$p$: Absolute pressure (Pa)
$p_0$: Standard atmospheric pressure (Pa)
$p_\text {in}$: Pressure in the right piston chamber (Pa)
$p_\text {out}$: Pressure in the left piston chamber (Pa)
$p_L$: Pressure in the left piston chamber (Pa)
$p_R$: Pressure in the right piston chamber (Pa)
$q_{a,e,p,L}$: Poiseuille flow rate of the axial external leakage (m^3^/s)
$q_{a,i,p,L}$: Poiseuille flow rate of the axial internal leakage (m^3^/s)
$q_0$: Temporary flow rate through the gap (m^3^/s)
$q_1 , q_2$: Temporary flow rate through the orifice (m^3^/s)
$q_{\text {out}}$: Dynamic output flow rate of the pump (m^3^/s)
$q_{\text {out,test}}$: Tested output flow rate (m^3^/s)
$\rho _0$: Oil density at atmospheric pressure (kg/s)
$S_{\text {in},L}$: Suction flow area of the left piston chamber ($\hbox {m}^2$)
$S_{\text {out},L}$: Discharge flow area of the left piston chamber ($\hbox {m}^2$)
$$s_a$$: Axial displacement (m)
$$t$$: Time (s)
$t_0$: Initial time of an arbitrary cycle (s)
$T$: Period of one complete pump rotation (s)
$$\text {Re}$$: Reynolds number
$\text {Re}_\text {crit}$: Critical Reynolds number for the transition from laminar to turbulent flow
$$v_a$$: Axial velocity (m/s)
$V_d$: Trapped volume of the piston chamber ($\hbox {m}^3$)
$V_g$: Displacement of the pump ($\hbox {m}^3$)
$V_L$: Volume of the left piston chamber ($\hbox {m}^3$)
$V_{\text {out}}$: Volume of the discharge chamber ($\hbox {m}^3$)
$\alpha$: Gas volume fraction
$$\delta _c$$: Gap height (m)
$\kappa$: Polytropic index of air, taken as 1.15
$\lambda _{\text {crit}}$: Critical flow number for the transition from laminar to turbulent flow
$\mu _0$: Dynamic viscosity of the hydraulic oil at normal temperature (Pa s)
$\omega$: Angular velocity around the axis of the piston shaft (rad/s)
$\varphi _c$: Maximum overlap angle (rad)
$\varphi _d$: Angle occupied by the dead angles (rad)
$\varphi _g$: Angle occupied by the distribution grooves (rad)
$\varphi _w$: Angle occupied by the distribution windows (rad)
$\theta$: Rotational angle (rad)
$\theta _0$: Distribution groove width angle (rad), which is $\pi$/2
$\eta _{v,\text {th}}$: Theoretical volumetric efficiency
$\eta _{v,\text {test}}$: Tested volumetric efficiency
